# Burden of idiopathic inflammatory rheumatic diseases in occupational healthcare: increased absenteeism and healthcare resource utilization

**DOI:** 10.5271/sjweh.4095

**Published:** 2023-07-01

**Authors:** Liisa Ukkola-Vuoti, Antti Karlsson, Samuli Tuominen, Mariann I Lassenius, Jaakko Aaltonen, Martta Ranta, Mikko Kosunen, Mari Renlund, Anne Lehtonen, Kari Puolakka

**Affiliations:** 1 Medaffcon Oy, Espoo, Finland.; 2 Terveystalo Biobank and Clinical Research, Turku, Finland.; 3 AbbVie Oy, Helsinki, Finland.; 4 Helsinki Rheumatic Diseases and Inflammation Research Group, University of Helsinki, Helsinki.

**Keywords:** burden of disease, registry study, sick leave

## Abstract

**Objectives:**

Patients with idiopathic inflammatory rheumatic diseases (IIRD) often have decreased working capacity resulting in indirect costs. However, data on patients’ short-term sick leave has been limited. This retrospective cohort study evaluated the number and length of sick leave, including short-term leave, and occupational healthcare resource utilization (HCRU) of the working-aged patients with IIRD compared to controls.

**Methods:**

The data on sick leave and occupational HCRU were gathered from the electronic medical records of the largest occupational healthcare provider in Finland from January 2012 to December 2019. Employed patients with an IIRD (including rheumatoid arthritis, spondyloarthritis, psoriatic and enteropathic arthritis, juvenile arthritis, and reactive arthritis) with at least a 12-months follow-up were identified and compared to age-, sex-, and follow-up matched controls without IIRD.

**Results:**

Altogether 5405 patients with IIRD were identified and compared with an equal number of controls. The patients incurred approximately 2.5 times more sick leave than controls: 21.7 versus 8.5 days per patient year, respectively. Short-term sick leave was common: 83% of sickness absence periods of the patients lasted 1–9 days and represented 30% of the total absenteeism. Loss of productivity due to lost workdays was on average €4572 (95% confidence interval €4352–4804) per patient year. Occupational HCRU was approximately 1.8 times higher among IIRD patients than controls.

**Conclusions:**

Workers with an IIRD incur considerably more sick leave and use more occupational healthcare services than controls. Short sick leave not registered in national insurance registers constitute a significant portion of days off work among patients with IIRD.

Idiopathic inflammatory rheumatic diseases (IIRD) are a group of chronic conditions characterized by pain, stiffness, and impaired function in the musculoskeletal system (1). Although modern drug treatment can in many cases alleviate symptoms (2) and reduce the risk of structural damage in joints and the back (3, 4), many patients still suffer from pain and functional limitations. These can, among employed patients, result in impairment of working ability manifesting as reduced productivity while at work (presenteeism), sick leaves (absenteeism), and work disability pensions. Patients with IIRD need long-term treatment and use plenty of healthcare services. These include contacts with physicians and other healthcare providers, laboratory tests, imaging, hospital care, and drug treatment. Consequently, IIRD lead to substantial direct costs (5). Decreased working capacity results in loss of work productivity translating to indirect cost (6, 7).

Studies on patients’ productivity loss have utilized several methods to retrieve data on lost workdays (8). Sick leave data obtained from self-reports or standardized interview questionnaires are prone to recall bias, while registry data can be considered a more reliable source. In Finland and Sweden, several studies have utilized social insurance registries (9–17). However, this data is lacking information on short-term sick leave, as only paid sickness allowances are recorded, ie, sick leave longer than the waiting period enacted by law, which in Finland is nine work days. A few studies on rheumatoid arthritis have used electronic medical records (EMR) which include also limited data on short-term sick leave but have focused on indirect costs (18, 19). As far as we know, no data exists on the exact occurrence of short sick leave among patients with IIRD.

In Finland, the working population is entitled to occupational healthcare services (OHS), which are financed and arranged by the employer and mostly provided by private healthcare providers (20, 21). Employers must provide employees with access to medical care. At a minimum, this includes preventive care in OHS, but usually also primary care and even specialized care may be included (20). Obviously, workers with chronic diseases like IIRD consume more OHS than average workers (22, 23), but reliable data is limited.

This study aimed to fill knowledge gaps on sick leaves and occupational HCRU of patients with IIRD by using EMR of a large OHS provider. Data on matched employees with and without IIRD are compared.

## Methods

### Study design and source population

This non-interventional, retrospective registry-based study utilized EMR of Terveystalo, which is the largest nationwide private and OHS provider with an integrated EMR system in Finland. Terveystalo has approximately 300 clinics around Finland, and in 2019 the total number of physician contacts was 3.7 million, corresponding to approximately 15% of annual visits or contacts nationwide (24). The Terveystalo data lake integrates most electronic systems used in routine outpatient care. The EMR – including demographic characteristics, diagnoses, healthcare contacts, and sick leave – were utilized in this study, which ran from 1 January 2012 to 31 December 2019.

### Inclusion criteria and matching of controls

*Idiopathic inflammatory rheumatic disease patient cohort.* Patients were identified in the EMR by the International Classification of Diseases 10^th^ Revision (ICD-10) codes: M02* (reactive arthropathies), M05* (seropositive rheumatoid arthritis), M06* (other rheumatoid arthritis), M07* (psoriatic and enteropathic arthropathies), M08* (juvenile arthritis), M45 (ankylosing spondylitis), or M46* (other inflammatory spondylopathies). Patients with M05* and M06* were combined (rheumatoid arthritis), as were those with M45 and M46* (spondyloarthritis). Patients who were OHS customers at Terveystalo with >12-month follow-up were included in this study.

Each patient was followed from the index date (the first record of any of the inclusion diagnoses) until the end of study (31 December 2019), death or end of occupational healthcare customer relationship. Some patients had multiple, distinct OHS customer periods (~10% overall), which may be due to, eg, changing jobs, temporary unemployment or data entry errors. Multiple customer periods of the same patient with a maximum gap of three months in-between were merged into a single period. If the break between OHS customer periods was more than three months, only the first continuous period containing the IIRD diagnosis was included in the analysis.

*Control cohort.* A one-to-one birth year, sex, and follow-up length-matched control cohort of OHS customers without ICD-10-codes M02*, M05*, M06*, M07*, M08*, M45 and M46* was formed from the same data source. The OHS customer periods for controls were handled identically to patients. No subjects were chosen twice for the control group. The controls were followed starting from the index day of the corresponding patients until the end of study (31 December 2019), death or end of occupational healthcare customer relationship, whichever occurred first.

### Outcomes

*Sick leaves.* The OHS provider prescribes and records sick leaves in the EMR. The total number and duration of sick leave periods (all-cause sick leave) and total sick leave days were obtained. Overlapping sick-leave periods were merged into one continuous period and counted only once. Subsequent, non-overlapping sick leave periods were counted individually. With respect to sick leave beginning prior to the index date or ending after the end of follow-up, only the days occurring during the follow-up period were counted.

*Sick leave costs.* The cost of lost productivity due to absenteeism was estimated. Other indirect costs were not accounted for. The human capital approach (25) was applied based on the age-group-specific median monthly salary in 2019 obtained from Statistics of Finland (26). Productivity cost per patient-year was calculated by multiplying one day’s productivity by the number of sick leave days divided by the follow-up time. To incorporate the supplementary social welfare expenses paid by Finnish employers, the annual salaries were multiplied by a factor as previously published by Martikainen et al (7). The multiplication factor for 2019 used in this study was 1.363, according to the official index of social welfare expenses determined by Statistics Finland (27).

*Occupational healthcare resource utilization.* Data on HCRU was retrieved from the EMR. Direct healthcare costs were calculated based on the contact type and length. For each HCRU type, the total number of events was collected and reported as the absolute value, per patient (total number of events/number of patients), and per patient year (PPY, total number of events/total follow-up). The point estimates are supplemented with 95% confidence intervals (CI) from bootstrapping (10 000 samples). HCRU data is presented per patient-year (cumulative number of events/cumulative follow-up).

*Occupational healthcare resource utilization costs.* Approximate costs of HCRU were obtained using the publicly available OHS provider’s 2020 price lists. In all cost analyses, overestimation was avoided, ie, if a specific HCRU item had multiple applicable prices, the lowest price was chosen. In our data source, a patient may have EMR which are either private or occupational contacts. However, as data on occupational/non-occupational contacts was not comprehensively available, all contacts were assumed to be occupational, which are generally less costly compared to private contacts (contacts outside of OHS). The types of contacts (nurse, general practitioner, specialist, physiotherapist, psychologist, etc.) were accounted for as well as the length of the contact as applicable. However, it was not possible to separate remote/online visits from face-to-face visits.

### Statistical analysis

For continuous variables, the mean, standard deviation (SD), median, first and third quartile points, and for all categorial variables, the numbers and proportions, were reported (table1). For analyses of differences between groups Kruskal-Wallis test (age) and Chi-squared test (sex) were used (table 1). Costs related to sick leaves and HCRU were calculated as total costs (grand total), per patient costs (grand total costs per total patients), and PPY costs (grand total of costs per grand total of patient years). CI were calculated using bootstrapping (10 000 samples). Sick leave costs corresponded to prices of the year 2019. In order to assess the association between patient characteristics ie, age, sex, IIRD subgroup, and patient–control status and sick leave and HCRU costs, cubic splines were fitted and plotted (costs ~ age). Separate splines were fitted in all patients per patient–control status for sick leaves and healthcare contacts separately. Only existing data were used, and no imputation of missing values was performed. Additional sensitivity analyses were not run. Data management and statistical analyses were performed using R version 3.6.1 on Ubuntu 18.04.3 LTS (28).

**Table 1:** Baseline characteristics of idiopathic inflammatory rheumatic disease patients and controls. M02: reactive arthropathies; M05/M06: rheumatoid arthritis; M07: psoriatic and enteropathic arthropathies; M08: juvenile arthritis ; M45/M46: spondyloarthritis; SD=standard deviation.

			All patients			M05/M06			M07			M08			M45/M46			M02	
			Patients (N=5405)	Controls (N=5405)		Patients (N=2008)	Controls (N=2008)		Patients (N=696)	Controls (N=696)		Patients (N=117)	Controls (N=117)		Patients (N=1638)	Controls (N=1638)		Patients (N=946)	Controls (N=946)
Follow-up time **per patient** (months)																			
	Mean (SD)		46.8 (24.6)	46.8 (24.6)		47.5 (25.2)	47.5 (25.2)		44.6 (23.8)	44.6 (23.8)		42.6 (26.2)	42.6 (26.2)		47.1 (24.2)	47.1 (24.2)		47.0 (24.1)	47.0 (24.1)
	Quartile																		
		25%	25.6	25.6		25.6	25.6		24.4	24.4		20.7	20.7		26.6	26.6		25.8	25.8
		50% ^a^	42.9	42.9		43.6	43.6		39.2	39.2		33.7	33.7		43.1	43.1		44.4	44.4
		75%	66.5	66.5		68.2	68.2		63.5	63.5		61.2	61.2		65.8	65.8		65.7	65.7
**Total** follow-up time years)			21 079.5	21 079.5		7948.3	7948.3		2586.8	2586.8		415.4	415.4		6429.2	6429.2		3705.2	3705.2
Age (years) ^b^																			
	Mean (SD)		45.4 (10.7)	45.4 (10.7)		48.1(10.2)	48.1 (10.2)		47.8 (9.6)	47.8 (9.6)		33.4 (10.7)	33.5 (10.7)		42.6 (10.5)	42.6 (10.5)		44.3 (10.8)	44.3 (10.8)
	Quartile																		
		25%	37.3	37.3		41.2	41.1		41.2	41.0		25.1	25.2		34.3	34.4		36.6	36.6
		50% ^a^	46.8	46.8		50.2	50.2		49.3	49.2		30.7	30.6		42.6	42.7		45.3	45.3
		75%	53.8	53.9		55.8	55.9		55.1	54.9		40.6	40.6		50.7	50.7		52.8	52.9
Sex (%)																			
	Female		59.3	59.3		73.0	73.0		49.1	49.1		76.1	76.1		50.3	50.3		51.1	51.1
	Male		40.7	40.7		27.0	27.0		50.9	50.9		23.9	23.9		49.7	49.7		48.9	48.9

^a^ Median. ^b^ At index.

Only pseudonymized data was used in the analyses, and data was published in aggregate such that an individual subject cannot be identified. All patients gave their informed consent for secondary use of health data to OHS provider in a format that the National Institute of Health and Welfare, Finland, has approved.

## Results

A total of 5405 OHS patients with an IIRD and a follow-up of at least 12-months were identified, and for each patient a randomly selected, age, sex, and OHS follow-up length-matched OHS control without IIRD was selected (supplementary material, www.sjweh.fi/article/4095, figure S1). Rheumatoid arthritis and spondyloarthritis were the most common diagnoses, representing 37% and 30% of the patients, respectively, whereas juvenile arthritis group represented only 2% (table 1). The mean age at index day was 45.4 years. As expected, differences in age and sex distributions were seen between diagnoses (P<0.001). Approximately 60% of all patients were female. The mean follow-up time was 3.9 years, and the total follow-up time was 21 080 patient years both in the IIRD patient and control cohort. The five most common co-diagnoses recorded during the OHS contacts were acute upper respiratory infections of multiple and unspecified sites (cases 43%, controls 33%; ICD-10 J06), dorsalgia (cases 30%, controls 21%; ICD-10 M54), acute sinusitis (cases 20%, controls 14%; ICD-10 J01), other joint disorders (cases 19%, controls 9%; ICD-10 M25), and other soft tissue disorders (cases 16%, controls 9%; ICD-10 M25).

### Number of sick leave days and duration of periods

**Figure 1:**
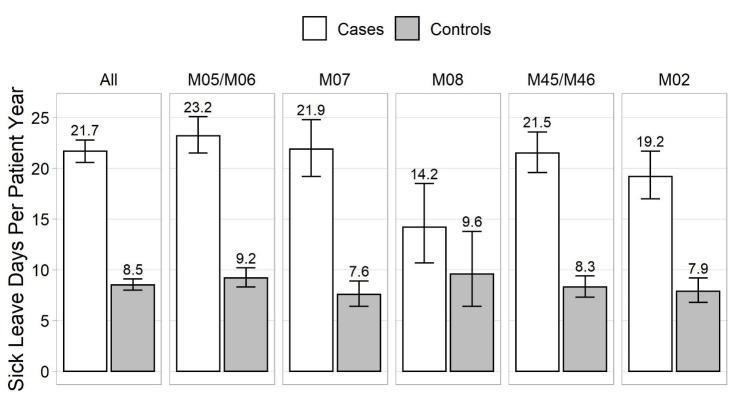
Number of sick leave days (with 95% confidence intervals) in employees with IIRD and their controls. M02: reactive arthropathies; M05/M06: rheumatoid arthritis; M07: psoriatic and enteropathic arthropathies; M08: juvenile arthritis; M45/M46: spondyloarthritis.

**Figure 2:**
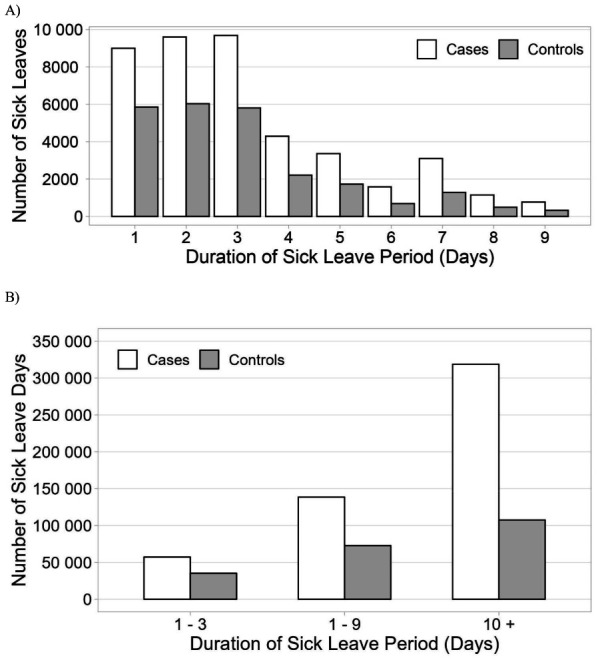
Number of sick leave periods by sick-leave duration (A) and cumulative number of sick leave days by sick-leave duration (B).

The number of sick leave days PPY is shown in figure 1. IIRD patients incurred markedly more sick leave days than controls: 21.7 versus 8.5 days, respectively. Compared to controls, the IIRD patients had almost double the number of sick leave periods: 2.4 and 1.3 PPY, respectively. The majority of sick leaves were short, the most common being ≤3 days (figure 2A). The cumulative number of sick leave days by sick leave duration is shown in figure 2B. We were especially interested in sick leaves lasting 1–9 days which, as a rule, are not eligible for sickness allowance and are not recorded in registry of the Social Insurance Institution of Finland. These represented 82.6% and 90.0% of sick leave periods and 30.3% and 40.4% of absenteeism among patients and controls, respectively (supplementary table S1).

### Occupational healthcare resource utilization

Patients with IIRD consumed 1.8-fold (ratio 95% CI 1.7–1.8) more OHS compared to controls (12.1 versus 7.1 contacts PPY, respectively). The most frequently contacted healthcare professional in both groups was occupational physician (3.7 versus 2.0 contacts PPY), followed by occupational general practitioner (1.8 versus 1.1 contacts PPY), laboratory visit (1.7 versus 0.9 visits PPY), specialist (1.6 versus 0.7 contacts PPY), and occupational nurse (1.4 versus 0.9 contacts PPY) (supplementary figure S2).

### Sick leave and healthcare resource utilization costs

Annual loss of work productivity due to sick leaves was €4572 (95% CI €4352–4804) per patient and €1807 (95% CI €1693–1929) per control (table 2). Respectively, the total occupational HCRU costs PPY were €621 (95% CI €607–636) and €353 (95% CI €342–364) (table 2). HCRU costs were lowest for the psoriatic and enteropathic arthropathies subgroup (M08) and highest for the juvenile arthritis subgroup (M07).

**Table 2:** Sick leave and occupational healthcare utilization number and costs (per patient year), and patient-control ratio for costs.

Resource type			Per patient year estimates			Patient–control ratio	
			Average	95% CI		Average	95% CI
Sick leave							
	Days (N)						
		Cases	21.7	20.6–22.8		-	-
		Controls	8.5	8.0–9.1			
	Cost (€)						
		Cases	4571.9	4351.7–4804.0		2.5	2.3–2.7
		Controls	1807.2	1692.7–1929.0			
Occupational healthcare resource utilization							
	Contacts (N)						
		Cases	12.6	12.34–12.86		-	-
		Controls	7.06	6.9–7.28			
	Costs (€)						
		Cases	621.0	606.8–635.7		1.8	1.7–1.8
		Controls	353.1	342.3–364.1			

**Figure 3:**
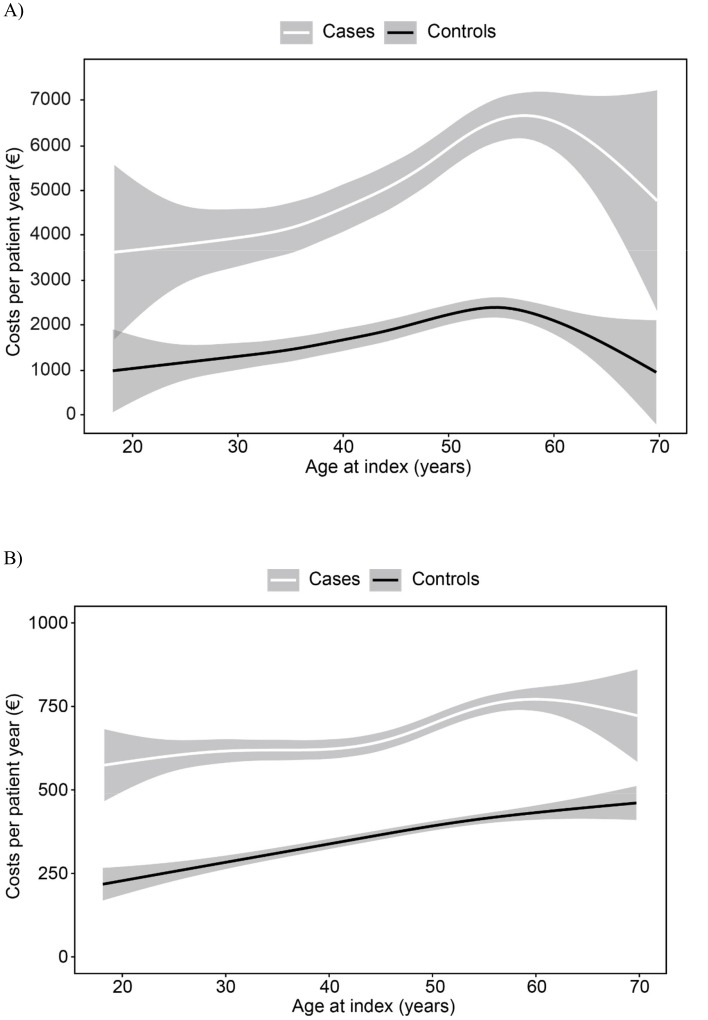
Costs per patient-year related to absenteeism (A) and occupational healthcare utilization (B) by age at the index date. Mean with 95% confidence intervals.

Productivity loss and OHS costs by age at the index date are illustrated in figure 3. The costs increased by age similarly for patients and controls but were consistently at a higher level among patients. The costs due to sick leaves were the highest slightly before the age of 60 years (figure 3A) and OHS contacts at the age of 60 (patients) or over (controls) (figure 3B).

## Discussion

The aim of this study was to fill knowledge gaps on work absenteeism and occupational HCRU of patients with IIRD. Our study demonstrated that the number of sick leave days and associated costs were 2.5 times higher for employees with IIRD compared to controls. The absenteeism was to a great extent (approximately 30%) due to short-term sick leave, which are not available from Finnish social insurance registers and have not previously been counted. Our data lacks some days off work because an employee’s self-notice is often enough for sickness absence lasting ≤3 days, and these very short sick leaves may not in all cases be recorded in EMR. However, approximately 60% of all sick leave periods in our study were ≤3 days long, which suggests a considerable coverage.

Short sick leaves are seldom comprehensively recorded in data sources worldwide. Few previous studies have been able to include data on short sick leaves of employees with rheumatic diseases (5, 18, 19), and none have analyzed the distribution of sick leaves by length or compared patients with controls without rheumatic diseases. The reported absenteeism has been at a similar level as in our study. A study from Germany reported 27 lost workdays within a year among patients with early RA (5), which is somewhat more than in our study (22 days). However, that study was conducted in 2001 before the development of more effective modern drugs, and absenteeism is more common in early than late RA. We had no data on the disease duration among our patients, but obviously it was longer than in the German study. A study from the US based on data from 2003 reported 21 absence days per year among employees with RA (19).

The number of days off work translates to productivity costs. Loss of productivity among patients with IIRD has been assessed by various methods in different populations (8). Some studies have used standardized questionnaires, while others have utilized registries to count the number of lost workdays. Human capital approach as well as friction cost method have been applied to estimate costs (25). Divergent social insurance systems also have impact on results, and comparison between studies is difficult. The reported lost productivity among patients with RA has been somewhat less (€1285 to €3292) than in our IIRD cohort (€4572) (5, 6, 29). Our estimate does not include permanent work disability.

The number of OHS contacts of patients was 1.7-fold compared to controls, with occupational physician being the most frequently recorded HCRU type by both. Occupational healthcare contact-related costs were 1.8-fold for patients with IIRD compared to controls. Our data, however, lacks the real drivers of direct health care costs, ie, medication or public healthcare resource utilization.

In our study, the costs of lost workdays and occupational HCRU increased with the employee’s age at the index day but starting reducing around the age of 60. This is intriguing because, in the general population, older age is associated with more disability and consumption of health services. An explanation might be that older employees are a kind of selected group, ie, those with poor health and limitations in function have already lost employment or ended up on disability pension. The general retirement age to earning-related pension in Finland was 63 years until 2017 and has been slowly increasing since then.

We reported all-cause sick leaves. Our data showed that also the diagnoses outside the inclusion criteria were more commonly recorded for patients with IIRD than for the controls. According to a recent Finnish study, in OHS the majority of sick leaves prescribed are due to musculoskeletal disorders, including arthrosis, dorsalgia, and joint disorders (30). The potential bias arising from short OHS follow-up periods was accounted for by excluding patients with follow-up lengths <12 months. The effect of seasonal infectious diseases is thereby ruled out, as well.

Our study has some limitations. The identification of employees with an IIRD was based on EMR of the OHS provider. In Finland, as a rule, rheumatologists or public rheumatology outpatient clinics diagnose IIRD. This is because the Social Insurance Institution (SII) requires a rheumatologist- or rheumatology clinic-issued certificate for a patient to be eligible for a special (higher) reimbursement of antirheumatic medication costs. This benefit is marked on the SII card, which every permanent resident of Finland has. Thus, patients with an IIRD are generally well aware of their diagnosis. The initial diagnosis date and the duration of the disease were not available in this study. In addition, with patient’s consent, the OHS providers can access electronic patient records of public healthcare. Furthermore, data for this study was retrieved from a large Finnish OHS provider. Thus, a selection bias towards specific job types is not expected. Note, however, that the OHS mainly has data on employed patients, and unemployed or retired patients will be more likely to be treated at public healthcare providers. The same applies to severe cases of IIRD. Thus, the findings of the current study can best be generalized to Finland and countries with similar healthcare systems that serve the employed and mild-to-moderate cases of IIRD. There was some apparent variation in the ICD-10-codes. Adult patients with juvenile arthritis (M08) were sometimes also given codes M05 or M06. In these cases, one record of M08 code was enough for a patient to be defined as having juvenile arthritis. Seropositive (M05) and seronegative rheumatoid arthritis as well as ankylosing spondylitis (M45) and spondyloarthritis (M46) were placed together. The observed typical differences in the patients’ age and sex distribution between different IIRD support the reliability of diagnoses. However, the date of diagnosis and consequently, disease duration of our patients is not known.

### Future recommendations for research, practice, and policy

The data source utilized in this study does not currently contain information on work disability pension, type of work (physical or non-physical), part-time employment, or unemployment in a structured format. Combining data with other real world data sources, such as nationwide registries on medications, pensions, other benefits/reimbursements, and linking patients’ data using the Finnish identification number would provide further possibilities for more detailed and wider studies on IIRD.

IIRD affect working-age individuals. Sick leave, HCRU, and morbidity burden highlight the importance of efficient follow-up to keep the patients with IIRD able to work. Optimal medical therapy and a personalized approach to risk stratification may provide solutions for this purpose. Despite advances in medical care, many patients still suffer from pain and functional restrictions, which may jeopardize their work ability (31). Also, non-pharmacological interventions can help keep IIRD patients employed (32). Healthcare professionals should endorse physical activity and other healthy behaviors implementing the EULAR recommendation of self-management strategies in IIRD (33). Preventive interventions before absence from work have been the most successful, and questionnaires may help identify workers at risk for sickness absence (34). Multiple short sick leave can serve as a “red flag” for problems to be addressed. There is a need for more studies on the impact of various non-pharmacological interventions on work ability outcomes.

Workplaces should consider the individual needs of workers with IIRD and develop and implement policies and practices to promote a workplace culture of inclusivity, flexibility and support (31). Part-time or suitable alternative work instead of sick leave may prevent the adverse consequences of staying at home. The patients’ emotional well-being should be assessed, self-esteem and self-efficacy supported and if necessary, advice given to them on OHS and/or other suitable services. Vocational rehabilitation aiming at work suitable for the patient should be started in case of permanent mismatch between requirements of work and capabilities of the patient. Targeted occupational HCRU can save productivity costs.

Policymakers, labor and trade organizations, and industry should be more involved in bridging the work participation gap between people with IIRD and the general population. Informed decision making requires robust and comprehensive data. Therefore, technologies that enable collection of structured registry data on fitness for work and adverse work outcomes should be developed and adopted.

### Concluding remarks

In the era of modern rheumatology, employed patients with IIRD still incur increased absenteeism and consumption of OHS. A substantial proportion of sick leaves are short and not captured in social insurance registers, thus they have been neglected in register studies. Employees with IIRD deserve special attention in occupational health care. Employers should implement practices that support their health and well-being at work.

### Statement of ethics and consent

The Terveystalo Biobank Research Ethics Committee approved this study (approval number: 20200311-A). All patients had given their written informed consent. All methods were carried out in accordance with relevant guidelines and regulations.

## Supplementary material

Supplementary material
