# Supplementary material for: Burden of idiopathic inflammatory rheumatic diseases in occupational healthcare: increased absenteeism and healthcare resource utilization
Source: Scand J Work Environ Health. 2023 Jun 30;49(5):341–9. doi: 10.5271/sjweh.4095 (PMC10713983; doi:10.5271/sjweh.4095)
Supplement: Supplementary material [file SJWEH-49-341-S001.pdf]

Burden of idiopathic inflammatory rheumatic diseases in occupational healthcare: increased absenteeism and healthcare resource utilization[1]

by Liisa Ukkola-Vuoti, PhD,[2] Antti Karlsson, PhD, Samuli Tuominen, MSc, Mariann I Lassenius, PhD, Jaakko Aaltonen, PhD, Martta Ranta, MSc, Mikko Kosunen, MSc, Mari Renlund, MSc, Anne Lehtonen, PhD, Kari Puolakka, MD, PhD

1. SUPPLEMENTARY DATA
2. Correspondence to: Ukkola-Vuoti, L.; Medaffcon Oy, Metsänneidonkuja 8, 02130 Espoo, Finland. [E-mail: liisa.ukkola-vuoti@medaffcon.fi

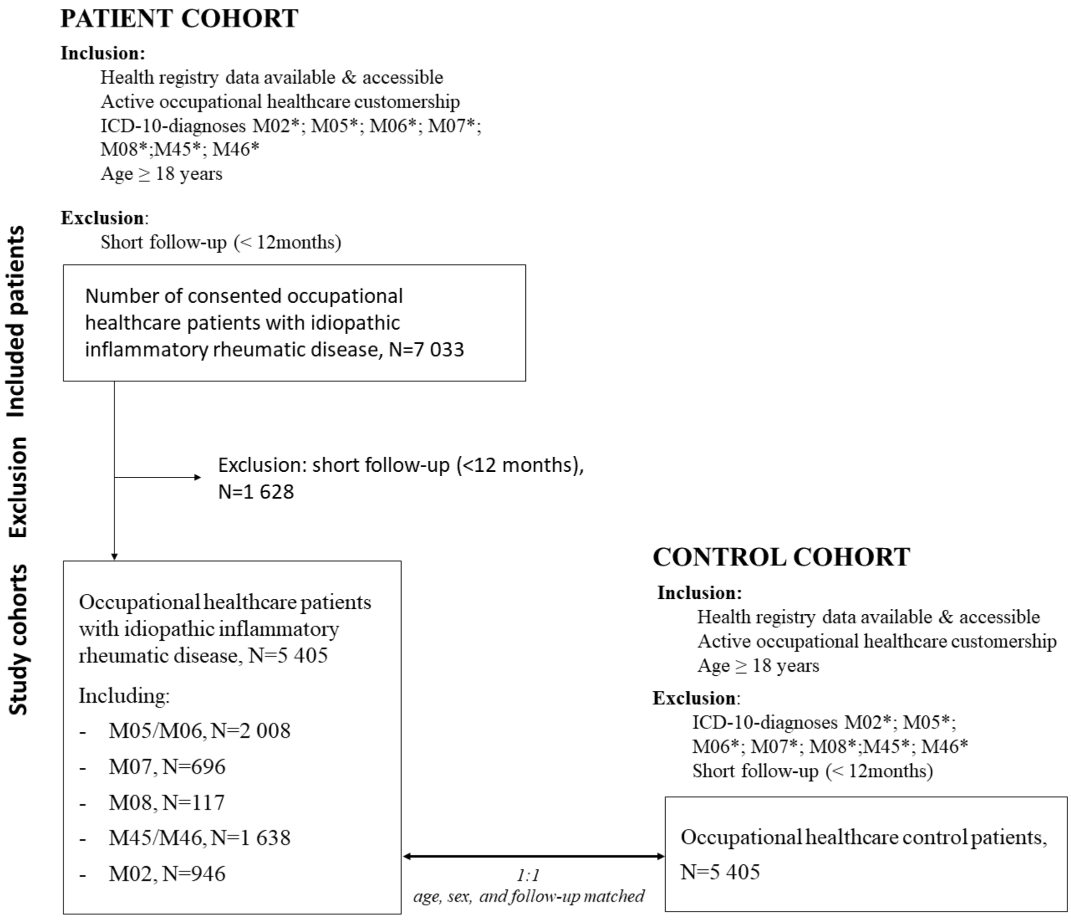

Figure S1. Flowchart of formation of the idiopathic inflammatory rheumatic disease patient and control cohorts. M02: reactive arthropathies; M05: seropositive rheumatoid arthritis; M06: other rheumatoid arthritis; M07: psoriatic and enteropathic arthropathies; M08: juvenile arthritis; M45: ankylosing spondylitis; M46: other inflammatory spondylopathies.

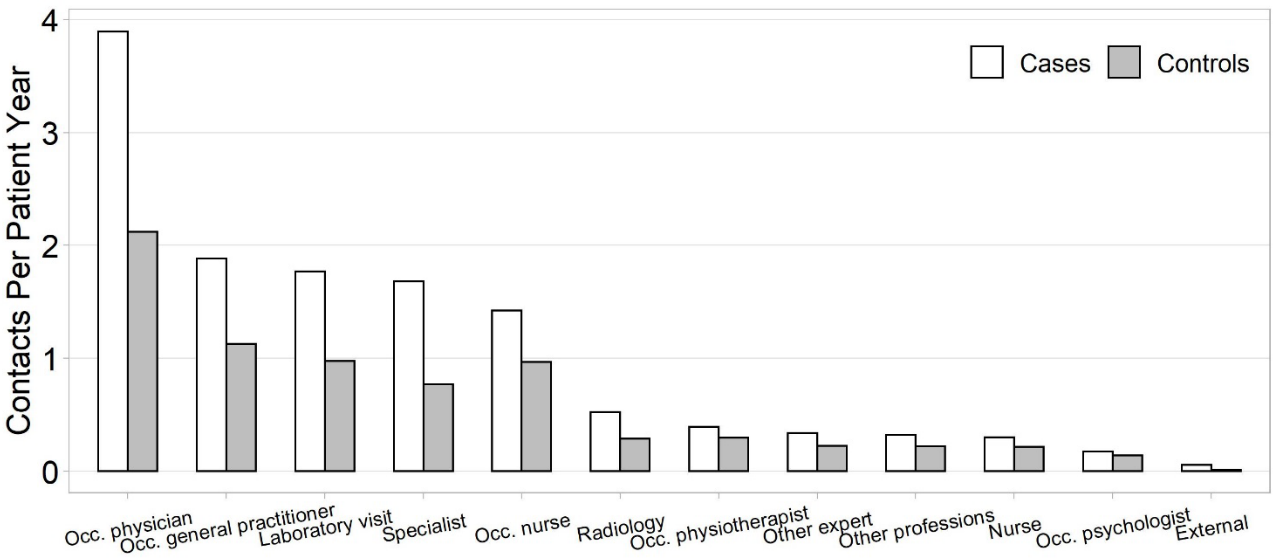

Figure S2. Healthcare resource utilization by type. Occ = occupational.

Table S1. Number and proportion of sick leave days and periods.

Cohort	Sick leave length (d)	Sick leave days (N)	Proportion of all sick leave days (%)	Sick leave periods (N)	Proportion of all sick leave periods (%)
**Patients**	**1 - 3**	57 277	12.5	28 294	54.9
**Patients**	**1 - 9**	138 542	30.3	42 545	82.6
**Patients**	**10 +**	318 718	69.7	8 959	17.3
**Patients**	**Total**	457 260	100	51 504	100
**Controls**	**1 - 3**	35 331	19.6	17 690	62.9
**Controls**	**1 - 9**	72 752	40.4	24 410	90.0
**Controls**	**10 +**	107 457	59.6	3 725	13.2
**Controls**	**Total**	180 209	100	28 135	100
